# Mini-mental state exam for children (MMC) in children with hemiplegic cerebral palsy

**DOI:** 10.1590/1980-57642016dn11-030011

**Published:** 2017

**Authors:** Ricardo Moura, Peterson Marco Oliveira Andrade, Patrícia Lemos Bueno Fontes, Fernanda Oliveira Ferreira, Larissa de Souza Salvador, Maria Raquel Santos Carvalho, Vitor Geraldi Haase

**Affiliations:** 1 Departamento de Processos Psicológicos Básicos – Instituto de Psicologia, Universidade de Brasília.; 2 Universidade Federal de Juiz de Fora, Departamento de Fisioterapia, Brazil, Departamento de Fisioterapia, Universidade Federal de Juiz de Fora – Campus de Governador Valadares., Universidade Federal de Juiz de Fora; 3 Pontifícia Universidade Católica de Minas Gerais. Programa de Pós-Graduação em Neurociências – Instituto de Ciências Biológicas, Universidade Federal de Minas Gerais.; Universidade Federal de Minas Gerais, Instituto de Ciências Biológicas, Programa de Pós-Graduação em Neurociências, Brazil, Universidade Federal de Minas Gerais; 4 Departamento de Ciências Básicas da Vida, Universidade Federal de Juiz de Fora – Campus de Governador Valadares.; 5 Programa de Pós-Graduação em Ciências da Saúde: Saúde da Criança e Adolescente – Faculdade de Medicina, Universidade Federal de Minas Gerais.; 6 Departamento de Biologia Geral – Instituto de Ciências Biológicas, Universidade Federal de Minas Gerais.; 7 Departamento de Psicologia – Faculdade de Filosofia e Ciências Humanas, Universidade Federal de Minas Gerais.

**Keywords:** cerebral palsy, Mini-Mental State Exam, screening, cognitive impairment, Paralisia cerebral, Mini-exame do estado mental, triagem, comprometimento cognitivo

## Abstract

Cognitive impairment is frequent in cerebral palsy (CP) and there is a lack of multiprofessional screening instruments.

**OBJECTIVE:**

The aim of this study was to investigate the utility of the Mini-Mental State Examination for Children (MMC), an adapted version of the Mini-Mental State Examination, in screening for cognitive impairments in children with CP.

**METHODS:**

We assessed 397 Brazilian children, 310 with typical development and 87 with CP (hemiplegic and quadriplegic forms), aged 5-16 years. Association between the MMC and general intelligence was assessed by the Colored Progressive Matrices instrument.

**RESULTS:**

Psychometric indexes for the MMC were adequate. ROC analyses revealed effective diagnostic accuracy in all ages assessed. Cut-off values are reported. Major difficulties on the MMC were observed in children with CP, particularly individuals with the quadriplegic form. Moreover, the MMC showed moderate correlation with the intelligence test, and was reliable in discriminating, among clinical cases, those with poorer cognitive abilities.

**CONCLUSION:**

The MMC could be useful as a multiprofessional screening instrument for cognitive impairment in children with hemiplegic CP. Results of the MMC in quadriplegic CP children should be interpreted with caution. Diagnosis should be confirmed by further psychological testing.

## RESUMO

Comprometimentos cognitivos são frequentes na Paralisia Cerebral (PC) e existe uma falta de instrumentos multiprofissionais para uma triagem.

**OBJETIVO:**

Investigar a viabilidade do uso do mini-exame do estado mental para crianças (MMC), uma adaptação do mini-exame do estado mental, como uma triagem para comprometimento cognitivo em crianças com PC.

**MÉTODOS:**

Nós avaliamos 397 crianças brasileiras, 310 com desenvolvimento típico e 87 com PC (hemiplégica e quadriplégica), com idades entre cinco e 16 anos. A associação entre MMC e a inteligência geral foi avaliada através das Matrizes Coloridas Progressivas de Raven.

**RESULTADOS:**

Os índices psicométricos para o MMC foram adequados. As análises ROC revelaram eficácia diagnóstica para todas as idades avaliadas. Os valores de corte são relatados. Dificuldades importantes na MMC foram observadas em crianças com PC, principalmente em crianças tetraplégicas. Além disso, MMC mostrou correlação moderada com o teste de inteligência e boa precisão na identificação das crianças com PC que possuem habilidades cognitivas prejudicadas.

**CONCLUSÃO:**

O MMC poderia ser útil como um instrumento de triagem multiprofissional para comprometimento cognitivo em crianças hemiplégicas. Os resultados de MMC em crianças tetraplégicas devem ser interpretados cuidadosamente. O diagnóstico deve ser confirmado por mais testes psicológicos.

## INTRODUCTION

Cerebral palsy (CP) is a heterogeneous neurological condition caused by congenital or early acquired focal or multifocal brain damage,^1^ and primarily defined by motor impairments in muscle tone and power. The underlying lesions are nonprogressive, but symptoms and functional impairments may progress, requiring early diagnosis, prevention and treatment.^2^ Although primarily defined by motor impairments, CP presents with a wider range of neurosensory, behavioral, and cognitive impairments,^3^^,^ 4 especially restrictive of adaptive functioning.^5^ These may vary from widespread intellectual disability to more circumscribed dysfunctions in language, executive functions, and school learning.^6^^,^ 7 The neuropsychological diagnosis requires IQ testing, and as neuropsychological services are in short supply, professionals need to establish referral criteria. Brief cognitive screening instruments could be used to identify patients in need of more thorough investigation, as has been done with considerable success in the case of aging-related dementing illnesses.^8^^-^ 10 Such brief screening instruments have been less successful in pediatric samples,^11^ mainly due to a lack of standardized measures,^12^ reliance on parental report,^13^ unknown correlations with general intelligence,^14^ requirements of motor dexterity,^15^ literacy,^16^ and lack of developmental sensitivity.^16^

There are numerous neuropsychological batteries used to assess children's cognitive function.^17^ These batteries usually are domain specific and require trained professionals for their application and long application times. There is a need for simple cognitive screening tests to allow the assessment of a wide range of cognitive domains in a short period. Such tasks could be a routine procedure, assisting in early detection of cognitive deficits.

The Mini-Mental State Examination (MMSE)^9^ was designed for screening cognitive dysfunctions, assessing the severity of impairments, and identifying changes over time. The MMSE is widely used for the evaluation of age-related cognitive decline,^8^^,^ 18 but it is still rarely used in the screening of developmental delays in childhood. In the study conducted by Jain and Passi (2005),^16^ a child-adapted MMSE showed brief implementation (5-7 minutes) in a wide age range (3-14 years). Moreover, comprehension of instructions was independent of socioeconomic status and educational level.

Pediatric versions of the MMSE have been used in Australia,^19^ India,^16^ Niger,^20^ Spain^21^ and USA.^22^ Preliminary investigations of Brazilian children suggest that the MMSE is useful for rapid assessment of children with CP.^23^^,^ 24 Nevertheless, these studies neither provide normative data for the MMSE nor assess psychometric properties of the MMSE for use in CP. Therefore, it is not known whether the MMSE can reliably discriminate the cognitive functions between healthy children and children with congenital focal and multifocal brain injury. Knowledge on MMSE accuracy in children with CP can contribute to the improvement of cognitive assessment by health professionals in primary care and neurorehabilitation centers.

In the present study we initially sought to determine the performance of typically developing (TD) children on the Mini-Mental State Examination for Children (MMC), an adapted version of the MMSE, in a preschool- and school-aged Brazilian sample. Subsequently, we examined the accuracy of the MMC in discriminating children with hemiplegic and quadriplegic forms of CP from TD children. Finally, in a subsample of TD and hemiplegic children, we investigated associations between the MMC and intelligence as well as its comparative accuracy for detecting cognitive impairment.

## METHODS

### Sample

We assessed 397 Brazilian children and youngsters aged 5-16 (mean=9.50, sd=2.15) years, from regular schools (Control Group; n=310) and neurorehabilitation centers (Clinical Group; n=87). For the Control Group, the inclusion criteria were regular school attendance and absence of parent-informed neurological dysfunction. The Clinical Group was composed of children diagnosed with spastic CP or stroke in early childhood. The Clinical Group consisted of 67 children with hemiplegic CP (Hemiplegia: Right n=35; Left: n=32), and 20 children with quadriplegic CP (Quadriplegia). Table 1 shows the sample sizes according to age and group. Data from all these children were used to analyze MMC accuracy.

**Table 1:** Sample sizes by age and group.

Age (years)	General sample				Subsample	
	Control (n=310)	Hemiplegia (n=67)	Quadriplegia (n=20)		Control (n=64)	Hemiplegia (n=49)
5-6	16	10	5		13	8
7	40	9	0		14	6
8	40	10	0		6	8
9	55	12	1		11	11
10	65	13	3		14	11
11	37	6	2		6	5
12	36	1	2			
13	15	3	3			
14-15-16	6	3	4			

Intelligence scores were available for a subsample of the Control and Hemiplegia groups (Table 1), comprising 64 (Control Subsample) and 49 (Hemiplegia Subsample) children, respectively. These data were used to investigate the concurrent and comparative accuracy of the MMC.

Informed consent was obtained in written form from parents and orally from children. Research procedures were previously approved by the local research ethics board (ETIC 250/09). For each participant in the study, a single investigator was responsible for the entire neuropsychological assessment.

### Assessment tools

*Mini-mental State Examination for Children (MMC).* A version of the Mini-Mental State Examination was adapted for children according to Jain and Passi.^16^ Preliminary versions of the MMC were prepared by two of the authors (PMOA and VGH). Choice of age-appropriate geometric figures were based on the Brazilian developmental neurological exam ("Exame Neurológico Evolutivo").^25^ The final version was decided consensually, comprising 13 items covering five cognitive abilities (orientation, attention and working memory, episodic memory, language and constructional praxis) with a maximum score of 37 (Appendix 1).

*Raven's Colored Progressive Matrices (CPM).* Fluid intelligence was assessed using Raven's Colored Progressive Matrices.^26^ Analyses were based on age appropriate z-scores calculated from the manual's norms.

### Statistical analyses

Analyses were conducted using R.^27^ One-way ANOVAs were conducted for group comparisons of continuous variables (age, MMC, CPM). ROC analyses were performed to verify MMC accuracy for discriminating the cognitive performance between Controls and children with CP. Cronbach's alpha was used to investigate internal consistency of the MMC scale and Pearson correlations were used to explore the associations between CPM and MMC. The significance level for all tests was 5%. To control for age differences, raw scores were converted to age-standardized z-scores.

The normality of scores was tested using the Shapiro-Wilk test, which revealed a non-parametric distribution for the MMC scores (W=0.63, p<0.001) and a parametric distribution for CPM scores (W=0.98, p=0.24).

## RESULTS

### Demographic characteristics

*Sex.* The study sample comprised 310 participants (53.9% female). A Fisher exact test indicated equal distribution of gender in the clinical and control groups (p>0.05).

*Age.* A one-way ANOVA with age in years as the dependent variable revealed that children with Quadriplegia (mean=10.60 years, sd=3.63 years) were significantly older than those in the Hemiplegia (mean=8.94 years, sd=2.23 years) and Control groups (mean=9.54 years, sd=1.97 years), F(2.394)=5.08, p<0.01, with small effect sizes (h^2^_*p*_=0.02). Post-hoc analyses, corrected for multiple comparisons with the Bonferroni method, revealed significant differences in age between Hemiplegia and Quadriplegia children only (p<.05). Importantly, age was similar across right and left Hemiplegia groups, F(1.65)=0.05, p=.82.

### MMC performance

*Group.* The Control Group showed the highest scores (mean=33.15, sd=3.63), followed by the Hemiplegia (mean=24.75, sd=7.12) and Quadriplegia (mean=3.85, sd=8.32) groups. All groups differed from each other significantly, F(2.394)=469.32, p<0.001, with a large effect size (h^2^_*p*_=0.70). Post-hoc comparisons revealed significant differences between all three groups (all p's<.001), with the Control Group having the highest scores, followed by the Hemiplegia and Quadriplegia groups in this order. Moreover, right and left Hemiplegia did not differ between each other, F(1.65)=0.51, p=.48.

For the subsample with intelligence testing, results showed no differences in age between Control and Hemiplegia groups (t=0.85, p>.05). Moreover, the Control subsample showed significantly higher scores on the MMC, (t=9.39, p<.001, d=1.94), and CPM scores (t=9.04, p<.001, d=1.70), as compared to the Hemiplegia subsample.

*Sex.* In the control group, small but significantly higher scores on the MMC were observed for female participants (mean_female_=33.6, sd_female_=3.27, mean_male_=32.6, sd_male_=3.92; t= –2.48, p<0.05, d=0.29). A similar but non-significant tendency was observed in clinical groups (mean_female_=21.4 sd_female_=9.23, mean_male_=18.5, sd_male_=13.39; all p's>0.05, d's=0.25). Considering the individual items, significant differences were observed only in the Orientation and Recall items, and with small to moderate effect-sizes (Cohen's d of 0.35 for Orientation, and 0.25 for Recall).

*Age.* MMC scores of the Control group increased with age (F=20.0, p<.001). Post-hoc analyses corrected for multiple comparisons showed significant increases in scores between 5-6 and 7-year-old children, and between 7-year-old and 8-year-old children (all p's<.001). MMC scores of children older than eight years tended to show the ceiling effect. For the clinical groups, the sample size was considerably smaller and age-related changes in MMC scores were small but significant (F=2.37, p<.05). Post-hoc analyses revealed no significant age differences in the clinical groups (all p's>.05).

### Psychometric characteristics of MMC

Internal consistency was acceptable, reaching a Cronbach's alpha of 0.78 for the Complete sample, 0.67 for the Control and 0.80 for the Hemiplegia group. The diagnostic accuracy of the MMC was investigated in the complete sample, in distinguishing all the clinical cases together (Hemiplegia and Quadriplegia groups) from the Control group, as well as the Hemiplegia and Quadriplegia groups separately. Table 2 presents the area under the ROC curves (AUCs), cut-off scores, specificity and sensitivity indexes, as well as positive and negative predictive power estimates.

**Table 2:** Diagnostic accuracy of MMC and CPM.

Sample		AUC	95% CI		Cut-off (z score)	Specificity	Sensitivity	ppv	npv
			Lower	Upper					
**a) Complete sample**	**MMC:** Clinical vs Control	0.919	0.888	0.951	–1.10	0.82	0.87	0.94	0.65
	**MMC:** Hemiplegia vs Control	0.896	0.856	0.936	–0.91	0.81	0.84	0.95	0.52
	**MMC:** Quadriplegia vs Control	0.993	0.981	0.999	–3.79	0.95	0.99	0.99	0.90
**b) Subsample**	**MMC:** Hemiplegia vs Control	0.916	0.862	0.969	–0.87	0.81	0.89	0.86	0.85
	**CPM:** Hemiplegia vs Control	0.884	0.824	0.943	0.62	0.84	0.78	0.86	0.74

* ppv: positive predictive value; npv: negative predictive value.

All lower confidence bounds for the AUCs were above 0.82. The comparison of the AUCs for the three ROC curves in the total sample indicated that the MMC was more accurate in distinguishing the Quadriplegia and Control groups (p<.01), mainly because 80% of the Quadriplegia group scored zero points. MMC accuracy in distinguishing Hemiplegia and Clinical groups from the Control group proved similar (all p's>.05). Both the MMC and CPM distinguished the Hemiplegia Subsample from the Control Subsample with lower bounds of accuracy higher than 0.82, but the performance of these two instruments did not differ (p>.05).

The best threshold of the MMC ROC curve in distinguishing clinical and control groups was set as a cut-off of 1 (z= –1.10, or a score of 28). As Table 3 shows, all Quadriplegia children scored below the cut-off of 1, with around 80% scoring no points on the MMC. We observed the opposite scenario when analyzing the Control group, as fewer than 2% scored below the cut-off score. In the Hemiplegia group, 30% of children scored below this cut-off.

**Table 3:** Cumulative percentiles and cut–off scores for the three groups on MMC.

MMC Score	General	Control	Hemiplegia	Quadriplegia
0	0.00	0.00	0.01	0.80
1	0.04	0.00	0.03	0.85
2	0.04	0.00	0.03	0.85
3	0.04	0.00	0.03	0.85
4	0.04	0.00	0.03	0.85
5	0.04	0.00	0.03	0.85
6	0.04	0.00	0.03	0.85
7	0.05	0.00	0.03	0.85
8	0.05	0.00	0.04	0.85
9	0.05	0.00	0.04	0.85
10	0.05	0.00	0.04	0.85
11	0.05	0.00	0.04	0.85
12	0.05	0.00	0.04	0.90
13	0.05	0.00	0.07	0.90
14	0.06	0.00	0.07	0.90
15	0.06	0.00	0.09	0.90
16	0.07	0.00	0.12	0.90
17	0.07	0.01	0.21	0.90
18	0.08	0.01	0.21	0.90
19	0.09	0.01	0.24	0.90
20	0.10	0.01	0.27	0.95
21	0.10	0.02	0.30	1.00
22	0.12	0.02	0.33	1.00
23	0.14	0.03	0.42	1.00
24	0.15	0.03	0.45	1.00
25	0.17	0.05	0.51	1.00
26	0.19	0.06	0.55	1.00
27	0.23	0.10	0.60	1.00
28	0.26	0.13	0.63	–
29	0.27	0.14	0.67	–
30	0.34	0.20	0.79	–
31	0.38	0.24	0.82	–
32	0.44	0.31	0.87	–
33	0.51	0.40	0.91	–
34	0.63	0.54	0.97	–
35	0.78	0.72	0.99	–
36	0.91	0.89	1.00	–
37	1.00	1.00	–	–

Due to the extremely low scores obtained by children with Quadriplegia, we calculated a second cut-off score, adjusted for the best threshold on the ROC curve that distinguished Control and Hemiplegia groups only. Considering Cut-off 2, the proportion of Hemiplegia children identified as positive cases increased to around 33%, with Raven's CPM scores 1 sd below controls.

Table 4 shows changes in mean scores, AUC and cut-off scores by age. The cut-off values were calculated based on the best balance between sensitivity and specificity values. The AUC was high, even for the age groups in which performance on the MMC approached a ceiling effect, with cut-off values higher than 32.

**Table 4:** Mean scores, AUC and cut-off values for each age level.

Age	Control				Clinical			AUC	95% CI		Cut-off score
	n	mean	sd		N	mean	sd		Lower	Upper	
5-6	16	26.94	3.02		15	12.73	9.85	96.88	92.05	100	24
7	40	29.80	4.36		9	19.89	8.48	89.17	78.19	100	25
8	40	33.80	2.72		10	24.90	5.74	95.25	89.88	100	31
9	55	33.16	3.47		13	25.08	6.76	86.85	76.36	97.35	32
10	65	34.83	2.07		16	24.38	12.53	90.48	80.77	100	33
11	37	34.30	2.27		8	21.25	13.32	95.78	90.29	100	32
12	36	34.61	2.43		3	11.33	19.63	91.20	73.36	100	35
13	15	33.80	2.46		6	15.67	15.63	86.11	66.90	100	30
14-15-16	6	31.83	3.92		7	14.57	11.47	95.24	84.13	100	30

### MMC and intelligence

**Figure 1:**
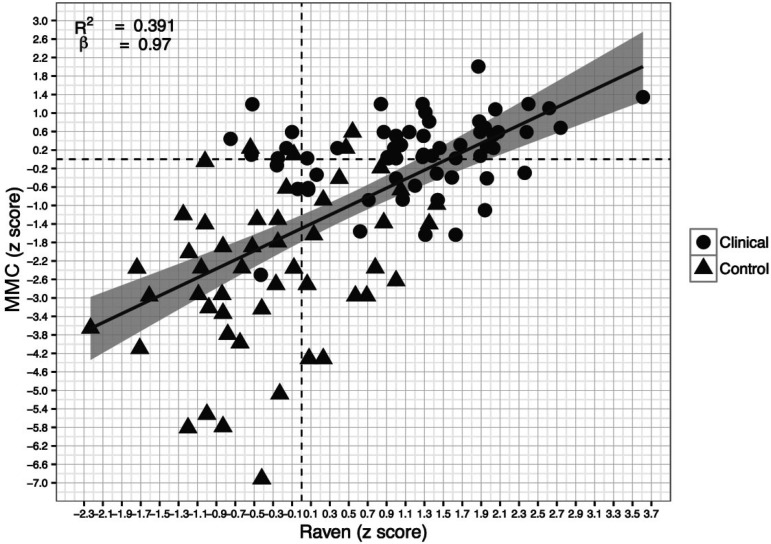
Scatterplot representing the correlation between the CPM and MMC, with dashed lines indicating the 0 point of each axis.

In this section, we investigated MMC scores in relation to intelligence, used here as a general index of cognitive functioning. MMC and CPM scores showed a moderate significant correlation (r=.62, p<.01). A scatterplot, with regression line, depicting the association between both tasks is shown in Figure 1. The intra-class correlation (ICC) also suggested acceptable agreement between measures (ICC=.54; 95%CI=.35-.66).

Figure 1 shows a higher concentration of clinical cases below the average score on the MMC (horizontal dashed line), with the region above the average score mostly occupied by control cases. When considering scores obtained on the CPM scale, the figure shows a similar pattern, as the region below the mean score is predominantly occupied by clinical cases. This suggests that both tasks were similarly effective in distinguishing clinical and control cases.

We tested whether the MMC is also powerful in discriminating, among hemiplegia participants, those at risk of more compromising cognitive deficits. Here, cognitive deficits were defined as a performance of 1.5 standard deviation below the mean (z <–1.5) on the CPM. To do so, we classified participants as positive cases (z <–1.5; n=4), and all other participants as negative cases (z ≥ –1.5; n=45). A ROC analysis revealed an AUC of 0.73 (95%CI=0.56 - 0.89), thus suggesting that the MMC may also discriminate cognitive abilities among clinical cases.

## DISCUSSION

To the best of our knowledge, this is the first study to report the use of the MMC, a modified version of the MMSE, in children and adolescents with CP. A PubMed search conducted in November 2016 yielded no articles for "mini-mental state examination AND cerebral palsy". Most previous studies compared the performance of a control group to that of children with mixed neurological impairments.^16^^,^ 19 ^,^ 22 We shall first discuss the association of MMC performance with demographic factors and CP. Next, we will analyze the psychometric properties of the MMC as a screening instrument for cognitive impairment in CP. Finally, we shall discuss the association of MMC performance with intelligence and its implications for screening of intellectual disability in CP.

### General factors associated with MMC performance

A slightly superior performance was observed in female participants. Although the clinical groups followed this trend, the differences were significant only in the Control Group. Previous research on the MMSE has not examined sex differences.^16^^,^ 19 ^,^ 21 ^,^ 22 A somewhat similar result was obtained in a longitudinal study.^28^ Rates of disappearance of neurological soft signs were higher for the female sex, especially at younger ages. This could reflect higher maturation rates of perceptual and motor functions in females.

Concerning age, MMC performance increased with age from preschool up to eight-year-olds. In the 9-year and older groups, performance on the MMC tended to plateau near the ceiling level. This pattern of age-related performance could also reflect developmental trends. In the Control subsample for which CPM data were available, although nonsignificant, correlations between this test and the MMC were lower for younger (r=.13) than for older children (r=.33). It could be hypothesized that MMC performance in younger children is more dependent on perceptual and motor abilities. Correlations between MMC and intelligence in the clinical groups were significant for both younger (r=.35) and older participants (r=.37).

Finally, performance of both CP groups was significantly lower than that of the Control Group, with lower performance in the Quadriplegia group than in the Hemiplegia group. MMC impairment in the Quadriplegia group was related predominantly to motor impairments (see below, section on psychometric characteristics).

### MMC as a screening instrument for cognitive impairment in cerebral palsy

MMC showed acceptable internal consistency, despite the tendency for the ceiling effect in scores. Concerning performance of Control Group children, on the one hand, the MMC proved to be an easy test, presenting no major difficulties for typically developing children. On the other hand, children with CP, especially the Quadriplegia group, showed more prominent difficulties, mostly related to motor impairments.

According to established interpretation criteria,^29^ high ROC accuracy estimates were observed for the discrimination of the Control Group from both Hemiplegia and Quadriplegia groups, as well as from the clinical cases as a whole. It should be noted, however, that MMC scores may underestimate cognitive skills of the Quadriplegia group, as performance on some items is highly dependent on motor skills. Therefore, motor deficits may be an important confounding factor for the assessment of these cases, and the high diagnostic accuracy reported here should be interpreted with caution. Parental reports could be an alternative to detect cognitive impairment in Quadriplegia children.^11^

### MMC as a screening tool for intellectual disability in cerebral palsy

Correlation between the MMC and CPM was significant and moderate-to-high. In the Hemiplegia group, none of the 49 children with scores of more than 1.5 standard deviations below the population mean on the CPM scored above the age-defined MMC cut-off score. This indicates the MMC may play a role in detecting cognitive impairments in children with Hemiplegia. Nevertheless, one should note that, as indicated by the ICC index, concordance between the MMC and CPM was not perfect. Specificities of each task, such as the higher reliance of CPM on abstract reasoning and the motor component of MMC, may explain why better concordance was not found.

Our results are consistent with the literature. MMSE scores are correlated with several educational attainment and IQ estimates in adult and aged samples of both brain-damaged and neurologically preserved individuals. Pearson correlation coefficients vary from 0.40 to 0.80.^30^^-^ 33

Some studies investigated correlations between scores for several versions of the MMSE and IQ in children. Ouvrier et al. (1993)^19^ observed correlations of 0.41, 0.83, and 0.79, respectively, for Full-Scale IQ, mental age and reading age. Another study observed lower correlations between MMSE scores and IQ: r=0.76 for mental age, and r=0.18 for verbal IQ. Results of the present study, showing a moderate correlation between MMC and IQ are consistent with the literature. Interestingly, we observed a correlation with a nonverbal estimate of intelligence, the CPM.

Our results suggest that MMC performance is influenced not only by general intelligence. As observed in the performance of the Quadriplegia Group, motor ability significantly influences MMC scores. We were not able to include school achievement measures in our study, an important factor influencing performance on the MMSE.^19^ The MMC could be useful for identifying Hemiplegia children with probable cognitive impairment. Further psychological testing should confirm diagnosis of IQ testing.

Limitations of the present study include the fact that CPM scores were not available for some participants. Also, our neuropsychological battery did not cover more specific cognitive abilities, such as working memory, visuospatial processing, and attention. Information about these abilities could allow more in-depth investigation into the cognitive basis of the MMC.

To conclude, the present study provided further evidence supporting the use of the MMC and abbreviated versions in clinical settings. In line with previous research, we also confirmed its utility in the neuropsychological assessment of CP.^23^^,^ 24 Specifically, the MMC was found to be a timesaving and reliable tool for investigating general cognitive abilities in CP children of different ages, available to a wide range of professionals in health services.

## APPENDIX 1

**Table:** MMC (Mini-Mental State Examination for Children) - Adapted version of the MMSE (Mini-Mental State Examination)

Function	Tests	Score
**Orientation**	Name, surname, age, sex	0-1-2-3-4
	Name of parents, state, city, place	0-1-2-3-4
	Age, month, day of month, day of week	0-1-2-3-4
**Object naming**	Pen, watch, glasses	0-1-2-3
**Digit span – forward**	5 -3	0-1-2-3-4
	4-7-2	
	5-9-3-1	
	2-7-5-9-4	
**Digit span – backward**	3-6	0-1-2-3
	2-9-5	
	4-1-9-7	
**Recall**	Pen, watch, glasses	0-1-2-3
**Naming body parts**	Naming body part indicated by the examiner: hand, foot, knee, nose, ear	0-1-2-3-4-5
**Command**	"Take the paper in your right hand, fold it in half, and put it on the floor" ("Pegue o papel com a mão, dobre-o ao meio e coloque-o no chão")	0-1-2-3
**Verbal string repetition**	"'No ifs, ands, or buts." ("Nem aqui, nem lá, nem acolá")	0-1
**Reading**	"Read this and do what it says" ("Close your eyes")	0-1
**Writing**	"Write your name"	0-1
**Constructional praxis**	"Copy the drawings. Do it as best you can" (Vertical line at age 3 years, cross at age 4 years, circle at age 5 years, square at age 6 years and diamond at age 7 years)	0-1
	**Maximum total score**	**37**
